# Breast cancer in the Thai Cohort Study: An exploratory case-control analysis

**DOI:** 10.1016/j.breast.2009.09.004

**Published:** 2009-10

**Authors:** Susan Jordan, Lynette Lim, Duangkae Vilainerun, Emily Banks, Nintita Sripaiboonkij, Sam-ang Seubsman, Adrian Sleigh, Christopher Bain

**Affiliations:** a School of Population Health, University of Queensland, Herston Road, Herston QLD 4006, Brisbane, Australia; b National Centre for Epidemiology and Population Health, ANU College of Medicine and Health Sciences, The Australian National University, Canberra, Australia; c Thai Health-Risk Transition Study, School of Human Ecology, Sukhothai Thammathirat Open University, Nonthaburi, Thailand; d Cancer Registry, Medical Informatics, Ramadhibodi Hospital, Mahidol University, Thailand

**Keywords:** Breast cancer, Etiology, Height, NIDDM, Birth rank, Low-incidence, TCS, Thai cohort study, STOU, Sukhothai thammathirat open university, NIDDM, Non-insulin dependant diabetes mellitus, BMI, Body mass index, OR, Odds ratio, CI, Confidence interval, PC, Personal computer

## Abstract

Breast cancer incidence may be increasing in Thailand but very little research has assessed core breast cancer risk factors in this country.

We used baseline questionnaire data from a national cohort study of Thai Open University students in an exploratory case-control study of breast cancer. The study included 43 female cases and 860 age-matched controls selected from the remaining 47,271 female cohort participants. Odds ratios and 95% confidence intervals were calculated using conditional logistic regression.

The women were predominantly premenopausal. Taller women had an increased risk of breast cancer (OR = 2.3, 95% CI 1.1–4.8, for height ≥160 cm vs ≤154 cm) as did women with non-insulin dependent diabetes mellitus (OR = 8.4, 95% CI 1.7–41). Women with older siblings had a reduced risk of breast cancer compared to those firstborn (OR = 0.3, 95% CI0.2–0.7).

Although limited by small case numbers, our findings suggest substantial increases in breast cancer rates in Thailand could be expected in the future.

## Introduction

Although there have been positive health benefits associated with Thailand's rapid socio-economic development, some negative health consequences of Western-style disease patterns are also emerging. Breast cancer is one example of this. In Western terms breast cancer is still relatively uncommon in Thai women (age-standardised incidence rate estimated at 20.5/100000 women^1^), but incidence has increased significantly over the last decade.^2^ There is also some evidence of regional variation, or at least urban excess with, for example, the Bangkok cancer registry recording incidence rates around twice those of the registries in the north-east of the country.^1^

In response to this increase in incidence the Thai National Cancer Institute (NCI) is implementing programs for breast cancer control, including promoting breast self-examination. However, primary prevention, a challenge in all countries, is hindered by the limited data on risk factors in Thailand. To our knowledge only three studies investigating breast cancer etiology in Thailand have been published,^3–5^ but these reported on only a limited set of potential risk factors. One reported only on specific single nucleotide polymorphisms;^4^ a second considered only the relation with hormone replacement therapy;^5^ while the third investigated associations with body size and energy intake amongst women from the Khon Kaen province.^3^ While it seems likely that reproductive risk factors for breast cancer are similar in different populations,^6–9^ other factors which differentiate Thai women from Western women- eg body sizes, diets, and other aspects of rapid social change- are relatively unstudied.

To help redress the lack of risk factor information, we have conducted a case-control study of breast cancer, analysing associations with a large set of exposures. Our data derive from baseline records for the Thai Cohort Study (TCS), a national Open University cohort currently under study for health-risk transitions.^10^

## Methods

### The cohort

The TCS includes 87,134 students of the Sukhothai Thammathirat Open University (STOU) who completed a mailed baseline survey in 2005. They represent 44% of the enrolled students contacted ^10^ and include 47,314 women. Relative to the Thai population as a whole, the cohort is more urban (nearly 50% versus about 40%) and rather better educated (most cohort members have finished high school). However, they are no richer than the average Thai (most cannot afford on-campus full-time study) and represent well the national regions and ethnic groups.^10^ The cohort study and its 20-page questionnaire focussed on the Thai health transition and for this reason, and the relatively young ages of participants, did not target all standard chronic disease risk indicators. However, sufficient relevant variables were included to allow an exploratory cross-sectional analysis that could help predict future burdens from breast cancer as Thailand moves further into its lifestyle and disease transitions.

### Case-control study

The questionnaire listed 24 diseases (including breast cancer), and participants were asked to mark any ever diagnosed by a doctor. All women who reported a diagnosis of breast cancer were contacted by mail or by telephone and asked to confirm their self-report, and to further provide their age at diagnosis, specifics of diagnostic procedures and treatments, and where these occurred. A definite diagnosis was established on clinical grounds for 43 women. For each confirmed case, 20 female controls matched on year of age were chosen at random from the rest of the cohort.

A wide variety of variables were assessed for possible associations with breast cancer: sociodemographic (education, income, marital status, ethnicity, urbanity, region), reproductive (parity, age at first birth, breastfeeding), lifestyle (alcohol, tobacco smoking, diet, body size, physical activity) and personal (medical and family history, and birth order).

#### Reproductive factors

Women were asked how many children they had and, their age at their first birth. Breastfeeding history was collected for a woman's last birth only.

#### Lifestyle factors

Participants were asked if they had ever smoked cigarettes or consumed alcohol, but regular amounts were not captured. Dietary questions targeted the balance of traditional and modern eating practices and requested limited quantitative information about food such as fruit and vegetables, soy-based foods, milk and deep-fried foods. Women were asked to record their height and weight. The relationship between height and breast cancer was assessed in three categories based on approximate thirds in the control women. We calculated body mass index (BMI – weight (kg)/height (m)^2^) and categorised this using suggested cut-points for overweight and obesity amongst Asian populations.^11^

In separate analyses^12^ we found that time spent watching television or using a computer and frequency of housework or gardening provided the most discriminating estimates of activity, so these measures were used to assess the relation with physical activity.

#### Personal factors

We examined relations with selected reported diseases including diabetes mellitus, hypertension, heart disease, asthma and various other cancers. We did not specifically ask for family history of breast cancer, but used whether their mother had died of cancer (where breast cancer would be a fairly major contributor) as an approximation of family history.

Data analysis was initiated with simple univariate case-control comparisons and tables were examined for differences in distributions that warranted further consideration. The relatively small case numbers precluded extensive stratified analyses.

Conditional logistic regression retaining the matching on age was then used to assess relations of variables with breast cancer after adjustment for potential confounders. A core model was established, including ever having had children (yes/no), height (in cm) and income (in Baht < = 10,000; 10,001–20,000; > 20,000). Other variables of interest were added to measure their adjusted effects, with models kept as parsimonious as possible in light of the small case numbers.

Menopausal status was not recorded in the questionnaire so we used age as a proxy, and repeated analyses restricted to women 45 years and younger at diagnosis (The mean age of menopause in Thai women has been reported to be between 49 and 52 ^13,14^). Case women's ages at diagnosis ranged from 28 to 51 (median 39) and the age-restricted analyses excluded only two case women. The median time since diagnosis for the women with breast cancer was three years (range 0–16 years).

## Results

Among the 47,314 female respondents to the baseline TCS questionnaire, 43 women were confirmed to have breast cancer and these women were matched on birth year to 860 controls.

### Descriptive findings

We see in Table 1 that the controls represent the source population of the cases reasonably well; there are no significant case-control differences in the sociodemographic variables presented, other than a suggestion that women with breast cancer have higher levels of education and higher incomes. Among controls we see some aspects of the Thai health transition clearly: increasing urbanisation and higher levels of education compared to their mothers.

In Table 2 associations between breast cancer and reproductive, lifestyle, and personal factors are presented.

### Reproductive variables

There was no significant difference in the occurrence of breast cancer between women who had and had not had children (OR = 0.9, 95% CI 0.5–1.7 for ever versus never having children), nor was there a significant relation with number of births or the age a woman was when her first child was born (OR = 1.0, 95% CI 0.3–3.1 for those aged 30 or more at first birth versus those aged 25 or less). A similar result was seen for breastfeeding (of the youngest child) amongst parous women (OR = 0.8, 95% CI 0.2–3.1). The number of case women with only one birth, and thus complete breastfeeding information, was too small (*n* = 5) to examine the effects in this group alone.

### Lifestyle variables

#### Body size

The tallest women had a two-fold increase in risk of breast cancer (OR = 2.3, 95% CI 1.1–4.8, for height ≥160 cm vs ≤154 cm). Although there were no significant differences in breast cancer according to BMI, odds ratios were elevated in women who were classified as underweight or obese compared to women of normal weight (OR = 2.7, 95% CI 0.9–8.0 and OR = 2.0, 95% CI 0.9–4.4, respectively). When analyses were restricted to women aged 45 years or less at diagnosis (presumptively premenopausal), the association with underweight became stronger (OR = 3.4, 95% CI 1.1–10.7) and that with obesity weakened (OR = 1.8, 95% CI 0.7–4.6), although the interaction was not statistically significant.

#### Physical activity/sedentary behaviours

There was no significant association between breast cancer and the selected measures of physical activity/sedentary behaviour (OR = 0.7, 95% CI 0.3–1.3 for >2 h of TV watching or PC use versus ≤2 h; OR = 0.9, 95% CI 0.5–1.8 for housework on most days versus housework less regularly).

#### Smoking and alcohol

Very few women were smokers or had drunk alcohol and we found no relation between these factors and breast cancer occurrence.

#### Dietary factors

The occurrence of breast cancer was reduced in women reporting consumption of milk (OR = 0.3, 95% CI 0.1–0.5 for weekly versus less than weekly consumption) and increased in those reporting daily versus less than weekly consumption of soy products (OR = 3.2, 1.2–8.4). Intake of fruit and vegetables did not vary by case-control status.

### Personal factors

Women who were the second or subsequent child born in their families had a significantly reduced risk of breast cancer when compared to those first born. There was also very strong and significant relation between non-insulin dependant diabetes (NIDDM) and breast cancer (OR = 8.4, 95% CI 1.7–41) although this was based on a very small number of affected case women (*n* = 3). No other reported diseases showed any association, nor was there one between our measure of family history of cancer and breast cancer.

## Discussion

Compared to controls, the women with breast cancer in our study were more likely to be taller; to have been diagnosed with NIDDM; to be first born in their families; and to consume soy-based foods frequently and milk infrequently. The data also suggested that both underweight and obesity may be related to breast cancer, but those findings were not statistically significant.

This study is exploratory and the perspectives offered here bear in mind its notable limitations. The small number of cases implies limited statistical power. Accordingly the discussion focuses on the positive results observed, since few conclusions can be drawn from the null results. We examined multiple exposures thus increasing the possibility of chance findings. The cases are also prevalent rather than incident, with the inherent consequence of some recalled items being influenced by knowledge of the diagnosis. In general we did not conduct analyses of variables likely to be particularly affected by this (eg current mental state), but our findings with respect to recent behaviours require cautious interpretations. The other usual challenges to validity are less likely to be troublesome here: Table 1 shows well the lack of selection bias within the cohort; and while we do not have precise information on some standard risk factors for breast cancer, we have quite a deal that is relevant, and confounding is not usually a major issue with this disease.

The women in our study are relatively young and were mostly premenopausal at diagnosis and thus our findings may be less applicable to older women, but in low-incidence populations such as Thailand the peak incidence occurs in women between the ages of 40 and 55^1,15–17^ so it is likely that our results are relevant to the age group of Thai women currently at highest risk.

The main established reproductive risk factors for breast cancer are nulliparity, late age at first birth, early age at menarche and late age at menopause;^15,18^ while prolonged breastfeeding reduces risk.^19^ Our results are not in close accord with these observations, but are not sufficiently robust to suggest that having children is irrelevant to the risk of developing breast cancer in this population. However, breast cancer risk may be elevated in the years immediately following a pregnancy,^15^ so a protective effect may not be apparent in this younger group of women. Other studies of low-incidence populations in Southeast/East Asia have generally found nulliparity and late age at first birth to be related to increased breast cancer risk,^7,8,16,20^ although an inverse relation with breastfeeding has been seen less often,^7,8,21^ possibly because high levels of prolonged breastfeeding continue to be the norm. Two other studies of breast cancer in Thai women also found no case-control differences in parity,^4,5^ although their results may also have been affected by methodological problems. We did not have information on age at menarche or age at menopause so were unable to assess the effects of these factors in this cohort.

The most consistent patterns we have observed here relate to body size. We found the tallest women had a two-fold increase in risk of breast cancer compared to the shortest. The association between height and breast cancer may reflect the influence of early diet or growth factors on breast cancer development^22^ and is consistent with observations in nearby Asian countries^16,20,21,23^ and more generally.^11^ The one relevant Thai study did not find a link between height and breast cancer, but the proportion of taller women in their study population was much less, perhaps obscuring any influence of greater height.^3^

With respect to BMI, amongst this group of mostly premenopausal women, those who were underweight or obese had the strongest association with breast cancer, although the non-significant nature of the findings needs bearing in mind. With older women excluded the effect of underweight appeared to strengthen and that of obesity to attenuate. Overall most studies have found elevated BMI associated positively with breast cancer in post-menopausal women and inversely in premenopausal women ^11^. Studies of other Southeast/East Asian populations have also mostly found positive associations with higher BMI, although patterns across menopause have been less consistent. While several, including one Thai study,^3^ have found positive relations only in post-menopausal women,^16,21,24,25^ some have found no variation by menopausal status^8,9,23^ and most have not found an inverse association premenopausally.^8,9,16,21,23,25^ Furthermore, a comprehensive meta-analysis of published cohort studies of breast cancer found that while high BMI was associated with increased risk of only post-menopausal breast cancer in North American and European populations, elevated BMI was associated with increased risks of both pre- and post-menopausal breast cancer in studies conducted in the Asia-Pacific.^26^ Few studies have specifically investigated underweight, but one from Malaysia (also mostly of premenopausal women) reported that women with a BMI of less than 18.5 kg/m^2^ had more breast cancer, as did those who were overweight or obese.^8^ It is of course possible that the association we see with underweight amongst this group of women with prevalent breast cancers reflects weight loss associated with breast cancer treatment or progression.

We also found an inverse relationship between higher birth order and breast cancer risk. Such a link is biologically plausible given that those first born are exposed to higher levels of oestrogens in utero compared to those later born.^27^ However, evidence from other epidemiological studies has been inconclusive.^28^

A diagnosis of NIDDM was also significantly associated with breast cancer occurrence in this population, although based on only three affected case women. As the cases were prevalent it is possible that the association reflects more regular medical surveillance in these women. However, other chronic diseases such as hypertension and arthritis were no more likely to be diagnosed in cases than controls suggesting diagnostic bias is not so likely; and a recent meta-analysis supports this positive relation, perhaps indicating a link between elevated insulin or IGF-1 levels and breast cancer development.^29^

We did not observe any clear evidence of a relation between physical activity or sedentary behaviours and breast cancer risk, but the study was underpowered to detect the moderate differences in risk generally associated with these. Physical activity may also be less relevant to this younger group of women as most evidence suggests that physical activity decreases the risk of post-menopausal breast cancer only.^11^ Although we found significant associations between consumption of milk and soy-based foods and breast cancer our findings contrast with what has generally been found ^30,31^ and it may be that these patterns of consumption reflect dietary changes subsequent to diagnosis rather than causal associations. We are also unable to draw any substantive conclusions regarding other dietary patterns and risks of breast cancer in this population.

Our results require confirmation in prospective data sets with full arrays of relevant variables, and in older post-menopausal women. However, put into context with other studies of breast cancer undertaken in similar populations, our findings suggest a substantial increase in breast cancer incidence could be anticipated in Thailand, but also point to potential avenues for cancer control in the country. Average heights have been increasing over the past few decades in several transitioning Asian populations,^17,24^ probably as a result of improving childhood nutrition and health,^32^ Few data are available for Thailand but, amongst the female TCS participants (*n* = 47,314), the youngest group of women (15–19yrs) are on average 3 centimetres taller than the oldest group of women (50+yrs) and there is a highly significant trend of increasing height with decreasing age (*p* < 0.0001). Our results and those of others suggest therefore, that as the cohorts of taller women age in Thailand, breast cancer incidence will increase accordingly. Other secular changes, such as a decrease in the average age at menarche^33^ will also likely contribute to increasing breast cancer incidence in Thailand as has been seen in countries further along the socio-economic transition such as Japan^34,35^ and Hong Kong,^36^ Although not readily modifiable, such secular changes highlight a growing need for population-based breast cancer screening.

Other risk factors identified here may be more amenable to primary prevention. Increasing rates of obesity^37^ and related increases in NIDDM^38^ are being observed in Thailand and these trends are expected to continue. Our results suggest that one consequence will be increased breast cancer rates, particularly amongst older women, and indicate that weight control may provide an important opportunity for breast cancer prevention.

## Conclusion

Although rates of breast cancer amongst Thai women are relatively low at present, the findings we have presented here, along with those of others, suggest a substantial increase could be expected in the future.

## Conflict of interest

There are no conflicts of interest. The funding bodies had no role in the study design; the collection, analysis and interpretation of data; the writing of the manuscript; or the decision to submit the manuscript for publication.

## Ethical approval

The study has ethics approval from the Australian National University, the Sukothai Thammathirat Open University (STOU) and the University of Queensland. All participants gave informed consent to the study.

## Figures and Tables

**Table 1:** Distributions of sociodemographic factors among women with breast cancer and their age-matched controls in the TCS.

	Cases n (%) *N* = 43	Controls n (%) *N* = 860	
Location of residence			
Now			
Urban	24 (57)	525 (62)	*p* = 0.6
Rural	18 (43)	330 (39)	
Aged 10–12			
Urban	20 (48)	314 (37)	*p* = 0.2
Rural	22 (52)	538 (63)	
Region			
Bangkok	8 (19)	202 (24)	*p* = 0.7
Central/East	15 (35)	262 (31)	
North/NthEast	13 (30)	281 (33)	
South	7 (16)	103 (12)	
Education			
High school	14 (33)	336 (39)	*p* = 0.09
Diploma	12 (29)	245 (29)	
Degree	16 (38)	276 (32)	
Mother's education			
None	5 (12)	139 (16)	*p* = 0.22
Primary	27 (63)	569 (67)	
Secondary +	11 (25)	140 (17)	
Income			
≤10000	9 (21)	302 (36)	*p* = 0.09
−10,001 to 20,000	15 (36)	287 (34)	
>20000	18 (43)	248 (30)	
Married/partner			
No	12 (32)	260 (32)	*p* = 0.5
yes	25 (68)	564 (68)	

**Table 2:** Associations between reproductive, lifestyle and personal factors and risk of breast cancer.^a^

Factor	Cases *n* = 43 n(%)	Control *N* = 860 n (%)	Adjusted^b^ OR (95% CI)
Ever had children			
No	19 (44)	324 (38)	1.0
Yes	24 (56)	523 (62)	0.9 (0.5–1.7)
Number of children			
0	19 (44)	325 (38)	1.0
1	5 (12)	166 (20)	0.6 (0.2–1.6)
2	13 (30)	277 (33)	0.9 (0.4–2.0)
≥3	6 (14)	77 (9)	1.5 (0.5–4.0)
Age at first birthc,d			
≤25	13 (54)	261 (50)	1.0
26–29	6 (25)	141 (27)	1.0 (0.4–2.9)
≥30	5 (21)	117 (23)	1.0 (0.3–3.1)
Breast fed youngest childc,d			
No	2 (9)	53 (10)	1.0
Yes	21 (91)	455 (90)	0.8 (0.2–3.1)
Breastfeeding durationc,d			
Nil	2 (9)	53 (10)	1.0
≤4 months	7 (32)	235 (47)	0.3 (0.1–1.4)
>4 months	13 (59)	216 (43)	1.0 (0.3–3.5)
Height cm			
≤154	12 (29)	370 (44)	1.0
155–159	11 (26)	215 (25)	1.5 (0.7–3.5)
≥160	19 (45)	262 (31)	2.3 (1.1–4.8)
BMI (Thai)			
<18.5	5 (12)	47 (6)	2.7 (0.9–8.0)
18.5–22.9	18 (43)	475 (56)	1.0
23–24.9	7 (17)	162 (19)	1.3 (0.5–3.1)
≥25	12 (28)	162 (19)	2.0 (0.9–4.4)
Mother died of cancer			
No	41 (95)	811 (94)	1.0
Yes	2 (5)	49 (6)	0.4 (0.1–3.0)
Birthrank			
First born	21 (51)	239 (28)	1.0
Later born	20 (49)	609 (72)	0.3 (0.2–0.7)
NIDDM			
No	40 (93)	850 (99)	1.0
Yes	3 (7)	10 (1)	8.4 (1.7–41)
Alcohol^e^			
Never	22 (51)	378 (45)	1.0
Ever	21 (49)	468 (55)	0.8 (0.4–1.5)
Smoking^e^			
Never	39 (93)	774 (93)	1.0
Ever	3 (7)	55 (7)	0.7 (0.2–3.0)
Milk^f^			
<wkly	23 (55)	269 (32)	1.0
≥wkly	19 (45)	582 (69)	0.3 (0.1–0.5)
Soy^f^			
<wkly	10 (23)	269 (32)	1.0
Wkly- <daily	22 (51)	420 (49)	2.2 (1.0–5.1)
daily	11 (26)	162 (19)	3.2 (1.2–8.4)
Fruit and Veg			
<4 serves	21 (49)	376 (45)	1.0
≥4 serves	22 (51)	457 (55)	1.0 (0.5–1.8)
Watching television/using a computer			
≤2 h daily	28 (65)	477 (56)	1.0
>2 h daily	15 (35)	370 (44)	0.7 (0.3–1.3)
Housework			
< most days	23 (53)	408 (48)	1.0
Most days	20 (47)	445 (52)	0.9 (0.5–1.8)
Region			
Bangkok	8 (19)	202 (24)	1.0
Central/East	15 (35)	262 (31)	1.5 (0.6–3.6)
Nth/NthEast	13 (30)	281 (33)	1.6 (0.6–3.9)
Southern	7 (16)	103 (12)	2.0 (0.7–5.9)

a Calculated from conditional logistic regression models.

b adjusted for height (continuous), income (≤10,000, 10,001–20,000,>20,000 Baht), ever had children(yes/no).

c additionally adjusted for number of children.

d amongst parous women only.

e additionally mutually adjusted for each other (i.e. alcohol for smoking; smoking for alcohol).

f additionally mutually adjusted for each other (i.e. milk for soy; soy for milk).

## References

[1] Khuhaprema T., Srivatanakul P., Sriplung H., Wiangnon S., Sumitsawan Y., Attasara P. (2007). Cancer in Thailand volume IV 1998–2000.

[2] Sriplung H., Wiangnon S., Sontipong S., Sumitsawan Y., Martin N. (2006). Cancer incidence trends in Thailand, 1989–2000. Asian Pac J Cancer Prev.

[3] Rattanamongkolgul S., Muir K., Armstrong S., Sriamporn S., Vatanasapt V. (2002). Diet, energy intake and breast cancer risk in an Asian country. IARC Sci Publ.

[4] Sangrajrang S., Schmezer P., Burkholder I., Boffetta P., Brennan P., Woelfelschneider A. (2007). The XRCC3 Thr241Met polymorphism and breast cancer risk: a case-control study in a Thai population. Biomarkers.

[5] Ratanawichitrasin A., Reansuwan W., Ratanawichitrasin S., Bhodhisuwan K., Kongpatanakul S. (2002). Risk of breast cancer in post-menopausal women using hormone replacement therapy. J Med Assoc Thai.

[6] MacMahon B., Cole P., Lin T.M., Lowe C.R., Mirra A.P., Ravnihar B. (1970). Age at first birth and breast cancer risk. Bull World Health Organ.

[7] Iwasaki M., Otani T., Inoue M., Sasazuki S., Tsugane S. (2007). Role and impact of menstrual and reproductive factors on breast cancer risk in Japan. Eur J Cancer Prev.

[8] Norsa'adah B., Rusli B.N., Imran A.K., Naing I., Winn T. (2005). Risk factors of breast cancer in women in Kelantan, Malaysia. Singapore Med J.

[9] Wu M.H., Chou Y.C., Yu J.C., Yu C.P., Wu C.C., Chu C.M. (2006). Hormonal and body-size factors in relation to breast cancer risk: a prospective study of 11,889 women in a low-incidence area. Ann Epidemiol.

[10] Sleigh A.C., Seubsman S.A., Bain C. (2008). Cohort profile: the Thai cohort of 87,134 open university students. Int J Epidemiol.

[11] World Cancer Resarch Fund/American Institue for Cancer Research (2007). Food, nutrition, physical activity and the prevention of cancer: a global perspective.

[12] Banwell C., Lim L., Seubsman S.A., Bain C., Dixon J., Sleigh A. (2009). Body mass index and health-related behaviours in a national cohort of 87,134 Thai open university students. J Epidemiol Comm Health.

[13] Trivitayaratana W., Bunyaratavej N., Trivitayaratana P., Kotivongsa K., Suphaya-Achin K., Chongcharoenkamol T. (2000). The use of historical and anthropometric data as risk factors for screening of low mBMD & MCI. J Med Assoc Thai.

[14] Morabia A., Costanza M.C. (1998). International variability in ages at menarche, first livebirth, and menopause. World Health Organization collaborative study of neoplasia and steroid contraceptives. Am J Epidemiol.

[15] Kelsey J.L., Bernstein L. (1996). Epidemiology and prevention of breast cancer. Annu Rev Public Health.

[16] Yoo K.Y., Kim Y., Park S.K., Kang D. (2006). Lifestyle, genetic susceptibility and future trends of breast cancer in Korea. Asian Pac J Cancer Prev.

[17] Shen Y.C., Chang C.J., Hsu C., Cheng C.C., Chiu C.F., Cheng A.L. (2005). Significant difference in the trends of female breast cancer incidence between Taiwanese and Caucasian Americans: implications from age-period-cohort analysis. Cancer Epidemiol Biomarkers Prev.

[18] Key T.J., Verkasalo P.K., Banks E. (2001). Epidemiology of breast cancer. Lancet Oncol.

[19] (2002). Breast cancer and breastfeeding: collaborative reanalysis of individual data from 47 epidemiological studies in 30 countries, including 50302 women with breast cancer and 96973 women without the disease. Lancet.

[20] Tung H.T., Tsukuma H., Tanaka H., Kinoshita N., Koyama Y., Ajiki W. (1999). Risk factors for breast cancer in Japan, with special attention to anthropometric measurements and reproductive history. Jpn J Clin Oncol.

[21] Shu X.O., Jin F., Dai Q., Shi J.R., Potter J.D., Brinton L.A. (2001). Association of body size and fat distribution with risk of breast cancer among Chinese women. Int J Cancer.

[22] Okasha M., McCarron P., Gunnell D., Smith G.D. (2003). Exposures in childhood, adolescence and early adulthood and breast cancer risk: a systematic review of the literature. Breast Cancer Res Treat.

[23] Ng E.H., Gao F., Ji C.Y., Ho G.H., Soo K.C. (1997). Risk factors for breast carcinoma in Singaporean Chinese women: the role of central obesity. Cancer.

[24] Iwasaki M., Otani T., Inoue M., Sasazuki S., Tsugane S. (2007). Body size and risk for breast cancer in relation to estrogen and progesterone receptor status in Japan. Ann Epidemiol.

[25] Hirose K., Tajima K., Hamajima N., Takezaki T., Inoue M., Kuroishi T. (2001). Association of family history and other risk factors with breast cancer risk among Japanese pre-menopausal and post-menopausal women. Cancer Causes Control.

[26] Renehan A.G., Tyson M., Egger M., Heller R.F., Zwahlen M. (2008). Body-mass index and incidence of cancer: a systematic review and meta-analysis of prospective observational studies. Lancet.

[27] Maccoby E.E., Doering C.H., Jacklin C.N., Kraemer H. (1979). Concentrations of sex hormones in umbilical-cord blood: their relation to sex and birth order of infants. Child Dev.

[28] Park S.K., Kang D., McGlynn K.A., Garcia-Closas M., Kim Y., Yoo K.Y. (2008). Intrauterine environments and breast cancer risk: meta-analysis and systematic review. Breast Cancer Res.

[29] Larsson S.C., Mantzoros C.S., Wolk A. (2007). Diabetes mellitus and risk of breast cancer: a meta-analysis. Int J Cancer.

[30] Trock B.J., Hilakivi-Clarke L., Clarke R. (2006). Meta-analysis of soy intake and breast cancer risk. J Natl Cancer Inst.

[31] Moorman P.G., Terry P.D. (2004). Consumption of dairy products and the risk of breast cancer: a review of the literature. Am J Clin Nutr.

[32] Gunnell D., Okasha M., Smith G.D., Oliver S.E., Sandhu J., Holly J.M. (2001). Height, leg length, and cancer risk: a systematic review. Epidemiol Rev.

[33] Mahachoklertwattana P., Suthutvoravut U., Charoenkiatkul S., Chongviriyaphan N., Rojroongwasinkul N., Thakkinstian A. (2002). Earlier onset of pubertal maturation in Thai girls. J Med Assoc Thai.

[34] Nagata C., Kawakami N., Shimizu H. (1997). Trends in the incidence rate and risk factors for breast cancer in Japan. Breast Cancer Res Treat.

[35] Wakai K., Suzuki S., Ohno Y., Kawamura T., Tamakoshi A., Aoki R. (1995). Epidemiology of breast cancer in Japan. Int J Epidemiol.

[36] Wong I.O., Cowling B.J., Schooling C.M., Leung G.M. (2007). Age-period-cohort projections of breast cancer incidence in a rapidly transitioning Chinese population. Int J Cancer.

[37] Aekplakorn W., Hogan M.C., Chongsuvivatwong V., Tatsanavivat P., Chariyalertsak S., Boonthum A. (2007). Trends in obesity and associations with education and urban or rural residence in Thailand. Obesity (Silver Spring).

[38] King H., Aubert R.E., Herman W.H. (1998). Global burden of diabetes, 1995–2025: prevalence, numerical estimates, and projections. Diabetes Care.

